# Linked linear mixed models: A joint analysis of fixation locations and fixation durations in natural reading

**DOI:** 10.3758/s13423-016-1138-y

**Published:** 2016-09-09

**Authors:** Sven Hohenstein, Hannes Matuschek, Reinhold Kliegl

**Affiliations:** Department of Psychology, University of Potsdam, Karl-Liebknecht-Strasse 24–25, 14476 Potsdam, Germany

**Keywords:** Linear mixed model, Model linkage, Eye movements, Reading

## Abstract

The complexity of eye-movement control during reading allows measurement of many dependent variables, the most prominent ones being fixation durations and their locations in words. In current practice, either variable may serve as dependent variable or covariate for the other in linear mixed models (LMMs) featuring also psycholinguistic covariates of word recognition and sentence comprehension. Rather than analyzing fixation location and duration with separate LMMs, we propose linking the two according to their sequential dependency. Specifically, we include predicted fixation location (estimated in the first LMM from psycholinguistic covariates) and its associated residual fixation location as covariates in the second, fixation-duration LMM. This linked LMM affords a distinction between direct and indirect effects (mediated through fixation location) of psycholinguistic covariates on fixation durations. Results confirm the robustness of distributed processing in the perceptual span. They also offer a resolution of the paradox of the inverted optimal viewing position (IOVP) effect (i.e., longer fixation durations in the center than at the beginning and end of words) although the opposite (i.e., an OVP effect) is predicted from default assumptions of psycholinguistic processing efficiency: The IOVP effect in fixation durations is due to the *residual* fixation-location covariate, presumably driven primarily by saccadic error, and the OVP effect (at least the left part of it) is uncovered with the *predicted* fixation-location covariate, capturing the indirect effects of psycholinguistic covariates. We expect that linked LMMs will be useful for the analysis of other dynamically related multiple outcomes, a conundrum of most psychonomic research.

**Electronic supplementary material:**

The online version of this article (doi:10.3758/s13423-016-1138-y) contains supplementary material, which is available to authorized users.

## Introduction

In research on complex behavior we often have to choose one of several, possibly equally plausible outcomes as the dependent variable for our analyses. Often these outcomes occur in a predetermined sequence. Let us take the sample case of eye-movement control during natural reading as a starting point. Fixation location and fixation duration are two outcomes that have greatly contributed to our understanding of oculomotor and psycholinguistic processes during natural reading. Moreover, they are dynamically related with each other. To some degree their relation has been the topic of previous research. For example, fixation duration was modeled as a joint function of word properties and fixation location (e.g., Heister, Würzner, & Kliegl, 2012; Kliegl, Nuthmann, & Engbert, 2006). Conversely, fixation location (implied in saccade amplitude) was modeled as a function of word properties and fixation duration (Wei, Li, & Pollatsek, 2013). Hence, either variable has been treated as a dependent variable in one model and as a covariate in the other. In general, however, the dynamics between these two measures (or their redundancy) has not been analyzed. Rather given the validity of both variables as indicators of cognitive processing efficiency, the most common scenario is to report two separate analyses or only one of them.

In reading studies, *linear mixed models* (LMMs) are a well-established tool for modeling fixation durations (e.g., Kliegl, 2007) and fixation locations (e.g., Yan et al. 2014). They are an extension of linear models and describe the response as a combination of fixed effects (e.g., word frequency, predictability, and length) and random effects (e.g., due to variability between subjects and items). Rather than estimating the individual or item differences from the average (fixed effects), LMMs estimate variance components on the assumption that differences between subjects and items are independently and normally distributed.

Here we propose to extend LMMs for the analyses of multiple outcomes, that is for a joint analysis of fixation location and fixation duration that takes into account the predetermined order of their occurrence in the behavioral stream. Specifically, we introduce a novel approach that includes results of the first LMM for fixation locations as covariates in the second LMM for fixation durations. As this procedure *links* the second LMM with the first LMM we refer to this approach as linked linear mixed modeling. Before getting into the technical and statistical details of this approach, we briefly review some basic facts about eye-movement control and what we know about fixation locations and fixation durations in reading.

### Perceptual span

Eye movements are constrained by the physiology of the eyes. Visual acuity declines monotonically from the center of the retina (i.e., the fovea) to the parafovea and out into the periphery. Due to this acuity constraint eye movements bring visual information from areas of intererst of the visual field into the fovea where it can be processed with high resolution (e.g., Findlay & Gilchrist, 2003). In reading, the areas of interest are the words of the sentence. Eye movements comprise a sequence of saccades and fixations. Therefore, eye movements in reading allow for the study of *where* the eyes fixate (the fixation location) and *when* a new saccade is executed (the fixation duration). Due to the complexity of eye-movement patterns, numerous alternative measures can be derived from these two fundamental measures (Inhoff and Radach 1998). These measures are used for studying perceptual and cognitive processes during reading (Rayner 1998).

During reading, visual acuity is strongly modulated by attention which is allocated preferentially in the direction of reading, leading to a highly asymmetric perceptual span (McConkie and Rayner 1975) that is the area of text from which information is extracted during a fixation. Specifically, the perceptual span extends only about 15 letter in and 3 letters against the reading direction relative to the currently fixated letter. The span is even smaller (i.e., up to six letters in reading direction) as far as effects of are concerned that depend on recognizing individual letters. The limits of the perceptual span provide strong constraints for the programming of saccades.

### Fixation location

Most saccades are executed in the direction of the writing system and about a third move the eyes from the currently fixated word *n* to the next word *n*+1 (e.g., Heister et al. 2012; Rayner, 2009). Inherently, the saccade to the next word is programmed before it is fixated and therefore, fixation locations depend on parafoveal information of the upcoming word. Rayner (1979) showed that the *preferred viewing location* is between the beginning and the center of the word, if the next saccade is directed towards the word to the right. Consequently, fixation locations are farther away from the beginning of a word as its length increases. In addition, the fixation location varies with the distance of the last fixation location from the beginning of the currently fixated word (i.e., its launch site): Fixation locations are closer to the beginning of the word when the last fixation was further away (McConkie, Kerr, Reddix, & Zola, 1988; Radach & McConkie, 1998; Rayner, Sereno, & Raney, 1996).

McConkie et al. (1988) studied the joint influence of word length, launch site and the distance to the target word on fixation locations. They showed that the distributions of fixation locations for different word lengths and launch sites follow a normal distribution. Interestingly, the effect of word length almost disappeared when the distance to the target word’s center was used to predict fixation locations. Hence, center-based fixation locations can be modeled as a linear function of center-based launch sites. Furthermore, they argued that fixation locations are subject to a saccadic range error that results in deviations from the intended fixation locations. Finally, Krügel and Engbert (2010) discovered that the launch-site effect is strongly modulated by word skipping. If the word before the target is skipped, the distribution of fixation locations is shifted leftwards compared to nonskipping saccades.

Whereas there is clear evidence of low-level visual influences on fixation locations, effects of linguistic variables have been somewhat controversial in the past, but are becoming clearer and clearer in current and ongoing research. Such effects indicate an influence of foveal processing difficulty of word *n* and parafoveal preprocessing difficulty of word *n*+1 on saccade-target selection. Lavigne et al. (2000) showed that the fixation location on highly predictable words is significantly shifted towards the end of the words in comparison to words which are less constrained from the prior context. However, such a predictability effect was not found in other studies (Rayner, Binder, Ashby, & Pollatsek, 2001; Vainio, Hyönä, & Pajunen, 2009; Vonk, Radach, & van Rijn, 2000). Also the evidence for an effect of parafoveal word frequency is limited: Rayner et al. (2006) reported a marginal effect indicating that the eyes land further into high-frequent words compared to low-frequent words. Such an effect was also found for reading Uighur sentences. Moreover, fixation locations shifted towards the beginning of a word as its morphological complexity (i.e., the number of suffixes) increased, clearly suggestive of parafoveal linguistic effect on fixation location (Yan et al. 2014).

There is also strong evidence from reading of Chinese sentences (both simplified and traditional script) that high psycholinguistic processing difficulty of word *n*−1 (i.e., usually the word fixated when the saccade to word *n* is programmed) as well as high processing difficulty of word *n* (i.e., the saccade-target word on which the fixation location is observed) shifts fixation locations towards the beginning of the word (Kliegl, Yan, Shu, & Tsai, 2014; see also Wei et al., 2013, for similar results using saccade amplitude rather than fixation location as dependent variable). (By high processing difficulty we mean low word frequency, high character complexity, and low word predictability.) The results are in agreement with the proposal that the perceptual span adjusts dynamically to local processing difficulty with saccade targets selected accordingly (Risse, Hohenstein, Kliegl, & Engbert, 2014; Schad & Engbert, 2012).

### Fixation duration

Arguably, fixation duration is the most prominent measure of psycholinguistic processing difficulty. There is a large body of evidence for default effects of length, frequency, and predictability (e.g., Rayner, 1998, 2009) of fixated words: Fixation durations increase with length (e.g., Just & Carpenter, 1980) and decrease with frequency (e.g., Inhoff & Rayner, 1986) and predictability (e.g., Balota, Pollatsek, & Rayner, 1985; Ehrlich & Rayner, 1981). In general, these effects remain significant even under statistical control of the other effects, although for word length a reversal in the direction of the effect (i.e., a suppressor effect) has been reported for such partial effects (Kliegl et al. 2006). Recent work indicates that the word-length effect on fixation durations is driven by the number of letters rather than the spatial width of a word (Hautala, Hyönä, & Aro, 2011).

The influence of word properties on fixation duration is not limited to those of the currently fixated word *n*; properties of the preceding word *n*−1 and the upcoming word *n*+1 also affect fixation duration on word *n* (Kennedy, Pynte, Murray, & Paul, 2006; Kliegl et al., 2013). While lag effects of word *n*−1 can be explained in terms of ongoing processing, successor effects of word *n*+1 reflect parallel processing of multiple words (see also Kennedy & Pynte, 2005) with a non-canonical effect of word- *n*+1 predictability (i.e., longer fixations for highly predictable parafoveal words) across different languages (for a review see Fernández, Shalom, Kliegl, & Sigman, 2014).

Especially important for the present context is that, aside from word properties, fixation duration is also systematically related to its location within the word. Indeed, there is a counterintuitive result, known as the *inverted optimal viewing position* (IOVP) effect (Vitu, McConkie, Kerr, & O’Regan, 2001), that fixation durations in the center of words are longer compared to fixation durations at the beginning or end of words. The result is counterintuitive because one would expect less processing difficulty for center than edge locations, and this expected result of a u-shaped curve is indeed obtained in experiments of isolated word recognition. Under experimental control of fixation location, the word center is the *optimal viewing position* (OVP) and requires the shortest exposure durations for correctly recognizing a word at a pre-specified level of accuracy (O’Regan, Lévy-Schoen, Pynte, & Brugaillère, 1984).

There are at least two explanations for the IOVP effect. One explanation, supported by computational simulations, is that it is primarily due to *mislocated fixations* (Nuthmann, Engbert, & Kliegl, 2005, 2007). The assumption of the simulation is that a proportion of fixations at the word boundaries are not on the intended word, but are a result of saccadic overshoot or undershoot due to oculomotor errors. Thus, the actual fixation location is different from the intended fixation location. Obviously, such mislocated fixations are more likely to occur at the beginning or end than in the center of words. The simulation also assumes that internally a signal about the mislocation (e.g., the difference between programmed and executed saccade amplitude) is quickly available and immediately triggers the start of a new saccade program, resulting on average in shorter fixations at the beginning and end of words.

Vitu et al. (2007) proposed the alternative explanation for why fixation durations are prolonged when the eye is at an optimal position for word recognition. According to their *perceptual-economy* hypothesis, fixating a word triggers localization processes that estimate the fixation location relative to the word boundaries. Fixation locations near the word center are most efficient—in agreement with results from isolated word recognition—and therefore delay saccade onset. Thus, this account assumes that fixations last longer when the eyes are estimated to be at locations where a greater amount of information is anticipated.

The IOVP effect is a good example to illustrate the case where a dependent variable (fixation duration) is modeled as a function of a covariate (fixation location) that in a different context itself is used as the dependent variable. During natural reading of sentences or texts, fixation locations and durations strictly alternate and neither of them is under experimental control. Hence, depending on the theoretical context of an analysis, either may be appear as dependent variable or as covariate. As both measures potentially correlate with the same psycholinguistic text properties of the fixated and neighboring words, there is ambiguity whether fixation durations are directly influenced by these features or only indirectly via an effect on fixation location.

### Linked linear mixed models

Before describing our approach with linked LMMs to deal with the ambiguities of effects of correlated covariates in the context of multiple outcomes, we introduce the basic statistics of LMMs with a separate model for each outcome.

An illustrative example model for fixation locations with the two covariates launch-site distance and word length is specified as follows:

1 $$ x = \alpha_{0} + \alpha_{d} \cdot d + \alpha_{\ell} \cdot \ell + \mathbf Z_{x} \gamma_{x} + \epsilon_{x}\,,  $$

where *x* is the dependent variable, the fixation location. The *α*_(⋅)_ represent the fixed-effect coefficients. The first one, *α*_0_, is the intercept. There are two covariates: launch-site distance, *d*, and word length, *ℓ*. The corresponding coefficients are *α*_*d*_ and *α*_*ℓ*_. Furthermore, **Z**_*x*_ is the model matrix for the random effects, and *γ*_*x*_ are the random-effects coefficients.^1^ Finally, *𝜖*_*x*_ are the *residuals*, the part of the observations *x*, that cannot be explained by the model.

In a second model, fixation location changes its role from a dependent variable to that of a covariate for fixation duration. An example with the three covariates fixation location, word frequency, and word length is

2 $$ t = \beta_{0} + \beta_{x} \cdot x + \beta_{f} \cdot f + \beta_{\ell} \cdot \ell + \mathbf Z_{t} \gamma_{t} + \epsilon_{t}\,,  $$

where *t* is fixation duration (time). In this model, the fixed-effects coefficients are denoted with *β*. Frequency is represented by *f*. As in Eq. 1, *x* denotes the fixation location, and *ℓ* stands for word length. Of course, the second model includes random effects (**Z**_*t*_ *γ*_*t*_) and residuals (*𝜖*_*t*_) too.

In this standard approach of separated model fitting, the results of the fixation-location model are not used for the fixation-duration model. However, we can use the predictions of one model as a covariate in a second model such that the estimates of the second model depend on the ones of the first model.

In LMMs the observed variable is expressed as a sum of (a) fixed effects, (b) random effects, and (c) residuals where the sum of fixed effects and random effects are the *predictions* or *fitted values*. To illustrate how an LMM affords a decomposition of an observed variable into fitted values and residuals, we annotate the fixation-location model (Eq. 1) as

$$\begin{array}{@{}rcl@{}} \underset{{\text{observed values}}}{\underbrace{x}}= \underset{\hat{x}\text{{, fitted values/predictions}}} {\underbrace{\alpha_{0} + \alpha_{d} \cdot d + \alpha_{\ell} \cdot \ell + \mathbf Z_{x} \gamma_{x}}}+ \underset{\text{residuals}}{\underbrace{\epsilon_{x}}}, \end{array} $$

where $\hat x$ represents the predicted fixation location, such that

3 $$ x = \hat x + \epsilon_{x}\,.  $$

In *linked* LMMs, we do not use the observed fixation location as covariate for fixation duration directly, but rather include the predictions and residuals of the fixation-location model as two new covariates. Hence, the two models are fitted *sequentially* such that the result of the first model is used as a covariate in the second one. In this approach, the fixation-location model (Eq. 1) remains the same. The fixation-duration model, however, is different, as it includes the predictions ($\hat {x}$) and residuals (*𝜖*_*x*_) of (Eq. 1):

4 $$ t = \beta_{0} + \beta_{\hat{x}} \cdot \hat x + \beta_{\epsilon_{x}} \cdot \epsilon_{x} + \beta_{f} \cdot f + \beta_{\ell} \cdot \ell + \mathbf Z_{t} \gamma_{t} + \epsilon_{t}\,.  $$

This model estimates two coefficients instead of one that are related to fixation location, $\beta _{\hat {x}}$ for predicted fixation location $\hat x$ and $\beta _{\epsilon _{x}}$ for residual fixation location, *𝜖*_*x*_.^2^

$$\begin{array}{@{}rcl@{}} t &=& \beta_{0} + \beta_{\hat{x}} \cdot (\alpha_{0} + \alpha_{d} \cdot d + \alpha_{\ell} \cdot \ell + \mathbf Z_{x} \gamma_{x})\\ &&+ \beta_{\epsilon_{x}} \cdot (x - \alpha_{0} - \alpha_{d} \cdot d - \alpha_{\ell} \cdot \ell - \mathbf Z_{x} \gamma_{x}) + \beta_{f} \cdot f\\ &&+ \beta_{\ell} \cdot \ell + \mathbf Z_{t} \gamma_{t} + \epsilon_{t}\,. \end{array} $$

Residuals are random deviations that are not explained by the model and, therefore, the covariate *𝜖*_*x*_ represents deviations caused by additional, potentially unknown sources, among them, for example, oculomotor errors. Although the linked LMM (Eq. 4) has one more fixed effect than the ordinary LMM (Eq. 2), there is no new independent covariate as the observed fixation location in Eq. 2 is simply the sum of the predicted ($\hat {x}$) and residual fixation location (*𝜖*_*x*_) of the model (Eq. 1). Hence the linked LMM is not augmented with additional information in terms of additional observations, but rather with *structural information* provided by the fixation-location model and its ability to decompose the observed fixation locations.

### The present study

We apply the technique of linked linear mixed modeling to data obtained in natural reading experiments. Specifically, we evaluate whether a decomposition of the observed fixation locations into predictions and residuals improves the model fit for fixation durations, compared to the classical approach using the observed values as covariate. To this end, we focus on the IOVP effect in single-fixation durations. Our expectation is that predicted fixation locations represent intended fixation locations and residuals represent oculomotor error. If the IOVP is actually due to oculomotor error, the effect of the residuals on fixation durations should resemble the IOVP curve. Furthermore, the shape of the curve resulting from the predicted values should resemble the OVP curve and thereby reflect the influence of fixation location on fixation duration. Since the OVP effect is based on isolated words, optimal fixation locations in normal reading may not coincide with the word center.

## Method

The data used in the analyses are from the Potsdam Sentence Corpus (PSC) as reported in Heister et al. (2012). We provide basic information; for details we refer to Kliegl, Grabner, Rolfs, and Engbert (2004) and Kliegl et al. (2006). The reanalysis provides new information about the data without loss of effects reported previously. Moreover, a parallel analysis was carried out for data from Beijing Sentence Corpus and Taipei Sentence Corpus to ensure generalizability of the results (see Kliegl et al., 2014).

### Subjects

A total of 273 subjects who varied widely in age, size of vocabulary, and cognitive ability participated in the experiment. They were paid € 5 to € 7 or received course credits. Participants gave their informed consent prior to their inclusion in the study. Sessions lasted 45–60 minutes. All were native speakers of German with normal or corrected-to-normal vision.

### Material

The PSC comprises 144 German sentences, ranging from five to eleven words (M=7.9, SD=1.4). Word frequencies range from 0 to 25,153 per million where the average log frequency was 2.1 and the standard deviation 1.3. Predictability norms were collected in an independent study with 272 native speakers of German. Predictability was measured as the probability of predicting a word after knowing the preceding part of the sentence. Excluding the first word of each sentence, word predictability ranges from 0 to 1 (M=0.20, SD=0.28). Since predictabilities were submitted to logit transformation for the present analyses (see below), values of 0 and 1 were changed to values in (0,1) with the function

$$p \mapsto \left\{\begin{array}{ll} \frac{1}{2 n_{c}} & \text{if} p = 0\\ \frac{2 n_{c} - 1}{2 n_{c}} & \text{if} p = 1\\ p & \text{otherwise,} \end{array}\right. $$

where *n*_*c*_ = 83 represents the number of complete predictability protocols for each word in the corpus.

**Table 1:** Transformations of continuous variables

Variable	Untransformed	Transformation
Fixation location *x*	Number of characters	$\frac {x}{\ell + 1} - \frac {1}{2}$
Fixation duration *t*	Milliseconds	$\ln t$
Word length *ℓ*	Number of characters	1/*ℓ*
Word frequency *f*	Occurrences per one million words	$\log _{10}(f+1)$
Predictability *p*	Probability of prediction	1/2 logit(*p*)
Launch-site distance *d*	Number of characters	$\log _{2} d$

### Appratus and procedure

Single sentences were presented on the center line. Participants were seated in front of the monitor with the head positioned on a chin rest. Eye movements of four samples were recorded with an EyeLink I system (SR Research, Toronto) with a sampling rate of 250 Hz or an EyeLink II system with a sampling rate of 500 Hz. All recordings and calibration were binocular. Participants were calibrated with a standard nine-point grid for both eyes. They were instructed to read the sentence for comprehension and to fixate on a dot in the lower right corner of the monitor to signal the completion of a trial.

### Covariates and responses

#### Variable transformations

Dependent variables and covariates were transformed prior to inclusion in the LMMs; for details see Table 1. We transformed response variables to meet the assumption of normally distributed residuals. Covariates were transformed nonlinearly since their distributions were highly skewed. There are no assumptions in LMMs on the distribution of covariates. However, highly skewed covariates probably have high-leverage points and are less likely linearly related to the dependent variable (e.g., Ruppert, 2011). High leverage refers to a value on a covariate that is far from the mean of that variable. These points can have an effect on the estimation of regression coefficients. Furthermore, nonlinear transformations of a covariate serve to linearize the relation between dependent variable and covariate, at least to a large extent; they reduce the need for higher-order polynomials and overall model compelexity in general. In all analyses, covariates were centered on their mean such that the intercept corresponds to the mean of the dependent variable. As much as possible and reasonable we have used the same transformations across other studies and data sets as well.

Absolute fixation location (i.e., the letter in the word to which the fixation was assigned) was transformed to relative fixation location (i.e., divided by word length) to facilitate comparison of words of different lengths. In the following, with fixation location we always refer to *relative* fixation location. We log-transformed fixation duration, word frequency, and launch-site distance to reduce highly positive skew. For word length we used its reciprocal value. Since predictability values are defined as probabilities, we used the logit transformation $p \mapsto \ln \frac {p}{1-p}$, where *p* denotes the predictability described above. Furthermore, we scaled the logits with $\frac {1}{2}$ to create values that are comparable with probits.

#### Modeling fixation location

Fixation location on word *n* is measured as the index of the character where the word is fixated. As covariate, we used the centered relative fixation location, in the following referred to as *fixation location*. With this transformation, 0 corresponds to a fixation location on the character in the middle of the word (e.g., position 3 in a 5-character word) and $-\frac {1}{2}$ corresponds to the space preceding the word. The interpretation of the fixation location is straightforward, since negative and positive values indicate fixation locations on the first and second half of word *n*, respectively.

We modeled fixation location on word *n* using several word-related and oculomotor covariates. The three major word properties length, frequency, and predictability of both the fixated word *n* and the preceding word *n*−1 were used as continuous covariates. Furthermore, we included launch-site distance as a covariate. Finally, we also included skipping of word *n*−1 as a covariate due to the different distributions of fixation locations for skipping and nonskipping saccades. Depending on the position of the previously fixated word relative to the currently fixated word, incoming saccades can be categorized as either skipping or nonskipping saccades. Nonskipping refers to the cases in which the previous fixation was on word *n*−1; skipping of word *n*−1 means the previous fixation was on a word preceding word *n*−1. In the majority of skipping cases, the saccade moves the gaze from word *n*−2 to word *n*. In our data set, word *n*−1 was fixated in 73.3 % and skipped in 26.7 % of all trials.

We specified a treatment contrast for this categorical variable with the value of 0 for nonskipping and 1 for skipping. With this specification the regression coefficient associated with this variable corresponds to an estimate of the difference (in fixation location) between the skipping and the nonskipping trials. Since nonskipping trials are more common, this category was chosen as the reference level. Consequently, the estimates for the remaining covariates correspond to the nonskipping trials Kliegl (2007). Motivated by inspection of zero-order effect plots, we specified interactions between skipping and all remaining covariates.

#### Modeling fixation duration

Our analysis focused on single fixations in first-pass reading as dependent variable. A fixation was classified as a *first-pass fixation* if neither the currently fixated word (*n*) nor any word to its right ($n+1, n+2, \dots $) was fixated before. If a word received exactly one fixation (in the first pass), this fixation was labeled as a *single fixation*. Furthermore, we only used single fixations that were preceded and followed by forward saccades to have a consistent influence of properties of word *n*−1 and word *n*+1 for all fixations in the analysis.

We modeled fixation duration on word *n* using word properties and fixation location. We included word length, frequency and predictability of words *n*−1, *n*, and *n*+1, that is, for the preceding, fixated and next word, respectively. Typically, effects are strongest for word *n*, followed by lag effects of word *n*−1 and successor effects of word *n*+1 (Kliegl et al. 2006; Kennedy et al. 2013). We also included a second-order orthogonal polynomial for fixation location on word *n* to capture the negative-quadratic IOVP effect of fixation location on fixation duration.

### Statistical analysis

Inferential statistics were based on LMMs specifying subjects, sentences, and words as partially crossed random factors (Baayen, Davidson, & Bates, 2008; Kliegl, 2007). Effects were estimated with the lme4 package (version 1.1-11; Bates et al., 2015) in the R environment for statistical computing (version 3.2.2, 64-bit build; R Core Team, 2015). All LMMs were fitted with the maximum likelihood criterion.

We specified varying intercepts for all random factors. Furthermore, we included variance components corresponding to the main effects for subjects (details see below). In these models, variance components are assumed to be independent to reduce their complexity. The variance components for sentences and words were much smaller than those for subjects. Therefore, aside from their intercepts, we did not include varying effects for words and sentences to keep model complexity at a level supported by the data (Bates, Kliegl, Vasishth, & Baayen, 2015; Matuschek, Kliegl, Vasishth, Baayen, & Bates, 2015). See the supplement for more details on the variance components of the LMMs.

We report regression coefficients with the *t* statistic. Degrees of freedom are not known for *t* statistics of LMMs, but for large numbers of subjects, words, sentences, and observations, as in this study, the *t* statistic converges to the *z* statistic of the normal distribution. For all tests we apply the two-tailed criterion (|*t*|≥1.96), corresponding to a 5 % error criterion for significance.

### Visualization of results

In all analyses, the estimated effects were based on models with multiple fixed and random effects. Figures with *partial effects* computed from model parameters reproduce the estimated statistical effects; they allow for a straightforward interpretation of the results. All figures in this paper are based on partial effects created with the remef package (Hohenstein & Kliegl, 2015) and were programmed with ggplot2 (Wickham 2009); Hohenstein and Kliegl (2014) provides examples for use of partial effects.

## Results

### Fixation location

LMM results with fixation location as dependent variable are displayed in Table 2. The model was fit with subject-related variance components for all main effects. The mean fixation location in the nonskipping trials (intercept) is slightly below zero, replicating the preferred viewing location slightly before the word’s middle character.

**Table 2:** Results for fixation location including estimated regression coefficients together with the *t* statistic

	Estimate	*t*
(Intercept)	−0.055	−9.168
Skipping of word *n*−1	−0.077	−14.409
Launch-site distance	−0.117	−61.020
Length (word *n*−1)	−0.283	−11.176
Predictability (word *n*−1)	0.014	7.736
Frequency (word *n*−1)	−0.009	−4.321
Length (word *n*)	0.506	12.629
Predictability (word *n*)	0.009	5.203
Frequency (word *n*)	0.012	3.856
Skipping × launch-site distance	0.002	0.466
Skipping × length (word *n*−1)	−0.050	−1.965
Skipping × predictability (word *n*−1)	0.013	7.075
Skipping × frequency (word *n*−1)	−0.017	−8.239
Skipping × length (word *n*)	−0.230	−9.386
Skipping × predictability (word *n*)	−0.008	−4.453
Skipping × frequency (word *n*)	−0.013	−7.063

The interaction effects have been tested for ambiguities with possible nonlinear main effects (see supplement and Matuschek & Kliegl, 2015)

**Fig. 1:**
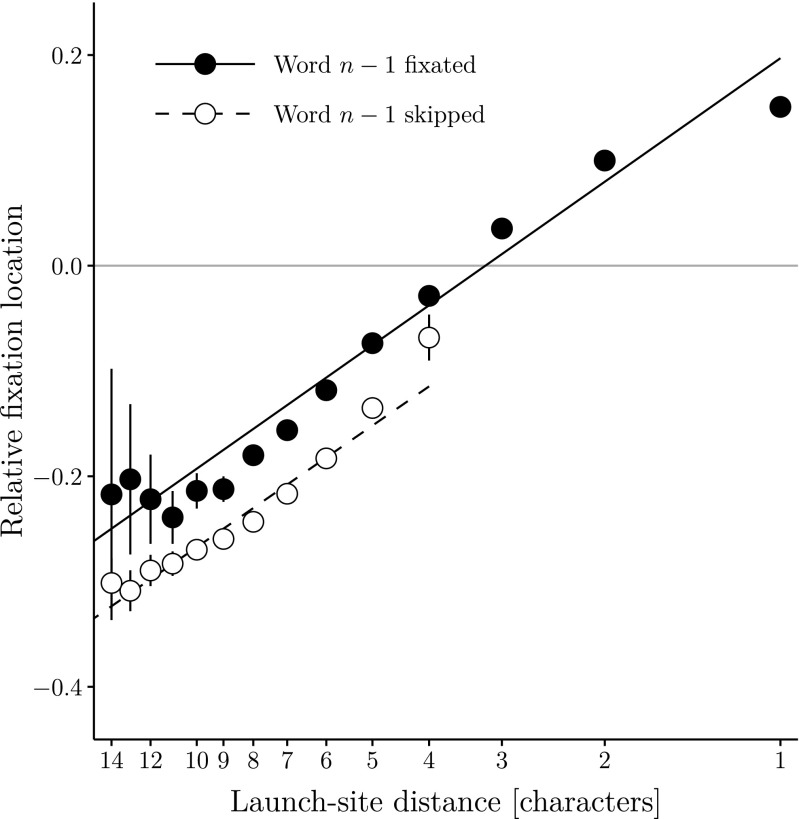
Average relative fixation location on word *n* as a function of launch-site distance (on a reversed log scale) together with linear regression lines. The figure displays partial effects created with the remef package (Hohenstein & Kliegl, 2015). Errorbars represent 95 % confidence intervals

Furthermore, there are considerable main effects of the oculomotor variables launch-site distance and skipping (see Fig. 1). As expected, fixation location decreases with launch-site distance: If a saccade starts from a position further away from the target word, the fixation location shifts to the left. The negative effect of skipping word *n*−1 indicates that the mean fixation location of a skipping saccade is further away from the word’s center as compared to saccades following the fixation of word *n*−1.^3^ There is no significant interaction between launch-site distance and skipping: The fixation location after a skipping saccade decreases with a slope that is not reliably different from the one of nonskipping saccades.

There are several significant main effects associated with properties of words *n*−1 and *n* (word length, frequency, and predictability) on fixation location on word *n*. With the contrast specification of skipping, coefficients of main effects correspond to estimates for nonskipping trials.

The length of word *n*−1 has a signifcant negative effect on fixation location. Since the word-length covariate is the reciprocal of the actual word length, the effects denote a leftward shift in mean fixation location for longer compared to shorter words *n*−1. As expected, the effect of the length of word *n* is reversed: Since the distance between launch site and target-word center increases with word length, fixation locations in longer words are further away from the center than in shorter words.

There are also reverse effects concerning word frequency. Whereas fixation location decreases with the frequency of word *n*−1, the opposite is true with respect to the frequency of the target word *n*. The positive effect of word *n* frequency indicates an influence of processing difficulty on fixation locations.

The effects of predictability of both word *n*−1 and word *n* are positive. As in the case with frequency, processing difficulty explains the right shift of fixation locations for high-predictable compared to low-predictable words. In contrast to word frequency, the predictability of the upcoming word depends on sentence context.

In addition to the main effects of word properties, corresponding to trials in which word *n*−1 was fixated before fixating word *n*, we find significant interactions with skipping of word *n*−1. This is not surprising, because in the majority of cases word *n*−1 corresponds to the *upcoming* word in skipping cases whereas it corresponds to the *fixated* word in nonskiping cases. For all interactions between skipping and properties of word *n*−1, the signs are identical to the signs of the corresponding main effects of word properties. Since both signs are equal, the effects of word *n*−1 properties are even stronger when this word is skipped than when it is fixated. Apparently, the computation of skipping saccades and thereby fixation locations strongly depend on the properties of the skipped word. Even if the word is not fixated directly, it shows its effect.

There is a different pattern for the interactions between skipping and the properties of the target word *n*. In contrast to word *n*−1, the regression coefficients of the interactions between skipping and word *n* properties and the ones of the corresponding main effects have opposite signs. For word length and predictability, the absolute value of the coefficients of the interactions are smaller than the ones of the main effects, indicating a weaker effect of these properties when word *n*−1 is skipped. With respect to word frequency, the coefficient of the main effect (0.012) is almost neutralized by the coefficient of the interaction (−0.0132). The sum of both coefficients (−0.00114) indicates the null effect of the target-word’s frequency on fixation locations for skipping trials.

In summary, the effects of word properties of the target word *n* on fixation location are stronger if word *n*−1 was fixated. In nonskipping trials, word *n* was closer to the preceding fixation because it was the immediate neighbor. Not suprisingly, more distant words (as in skipping cases) exert a weaker effect.

### Fixation duration

Here we analyse fixation durations on word *n* (focusing on the IOVP effect) as a function of several covariates including fixation location. We use both (a) the classical approach of *separate* models with observed fixation locations and (b) *sequential* linked LMMs.^4^

#### Separate models: Observed fixation location

Adding a second-order polynomial of *observed* fixation location to a model including only word-related covariates yields a significant improvement in goodness of fit for AIC and BIC criteria (see Table 3). As expected, we obtain several significant effects of properties of words *n*−1, *n*, and *n*+1 on the fixation duration on word *n*, as in previous analyses of subsets of these data Kliegl et al. (2006). The model coefficients are displayed in Table 4.

**Table 3:** Goodness-of-fit statistics of linear mixed models of fixation duration

model dependency	Fixation location covariate	df	AIC	BIC	log likelihood
separate	none	23	24014	24228	−11984
	observed	25	23842	24074	−11896
linked	predicted	25	20177	20409	−10063
	residual	25	23011	23243	−11480
	predicted and residual	27	18817	19068	−9381

All values are rounded to the nearest integer. “df” denotes “degrees of freedom”

**Table 4:** Results for fixation duration including estimated regression coefficients together with the *t* statistic

	LMM		Linked LMM	
	Estimate	*t*	Estimate	*t*
(Intercept)	5.303	471.953	5.304	471.098
Fixation location (observed, linear)	−2.644	−9.100		
Fixation location (observed, quadratic)	−2.903	−9.922		
Fixation location (predicted, linear)			−21.036	−65.833
Fixation location (predicted, quadratic)			1.697	5.837
Fixation location (residual, linear)			7.692	28.958
Fixation location (residual, quadratic)			−6.508	−23.091
Length (word *n*−1)	0.046	1.222	−0.134	−3.763
Predictability (word *n*−1)	−0.010	−3.649	−0.010	−3.561
Frequency (word *n*−1)	−0.029	−9.426	−0.032	−10.459
Length (word *n*)	0.130	1.624	0.202	2.488
Predictability (word *n*)	−0.029	−10.173	−0.028	−10.295
Frequency (word *n*)	−0.023	−3.686	−0.016	−2.461
Length (word *n*+1)	0.223	7.275	0.240	7.947
Predictability (word *n*+1)	−0.004	−1.310	−0.007	−2.440
Frequency (word *n*+1)	−0.017	−5.310	−0.013	−4.267

LMM with observed values (columns 2 & 3); linked LMM with predicted and residual values (columns 4 & 5)

With respect to the currently fixated word *n*, fixation duration decreases with predictability and frequency. The effect of (reciprocal) word length is positive but not significant. The same pattern is present for the properties of the preceding word *n*−1 and the next word *n*+1. For word *n*+1, the effect of word length is significant. If the upcoming word is shorter, fixation duration increases. With the exception of a significant word *n*+1 length effect, the results are in agreement with Kliegl (2007).

**Fig. 2:**
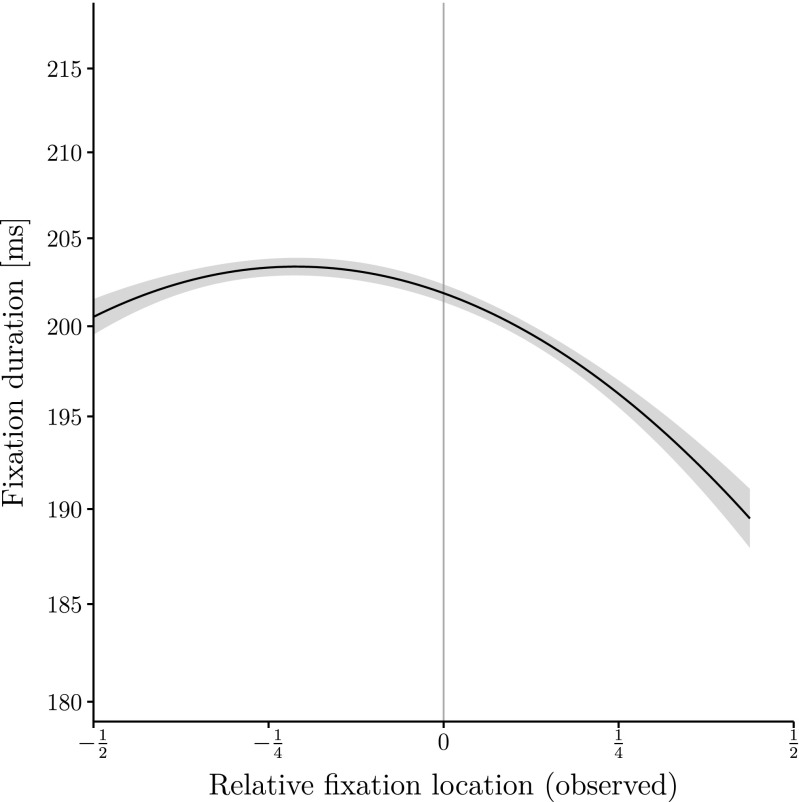
Fixation duration on word *n* (on a log scale) as a function of observed relative fixation location together with a second-order polynomial regression curve. The figure displays partial effects created with the remef package (Hohenstein and Kliegl 2015). Errorbands represent 95 % confidence intervals

Furthermore, both the linear and the quadratic trend of fixation location are significant. The quadratic effect is negative indicating an IOVP effect: Fixation duration is highest at the word’s center and decreases towards its edges (see Fig. 2). In summary, the classical approach of using observed fixation location for modeling fixation duration results in the expected outcome: the IOVP effect.

#### Sequential linked models: Predicted and residual fixation location

Here, rather than using observed fixation location for modeling fixation duration, we incorporate the results of the fixation-location LMM (see above). Specifically, this approach allows three different ways to include fixation location: (a) predicted fixation location, (b) residual fixation location, and (c) both predicted and residual fixation location. We evaluate the goodness-of-fit of these approaches and compare them with the one of the model using observed fixation location.

**Fig. 3:**
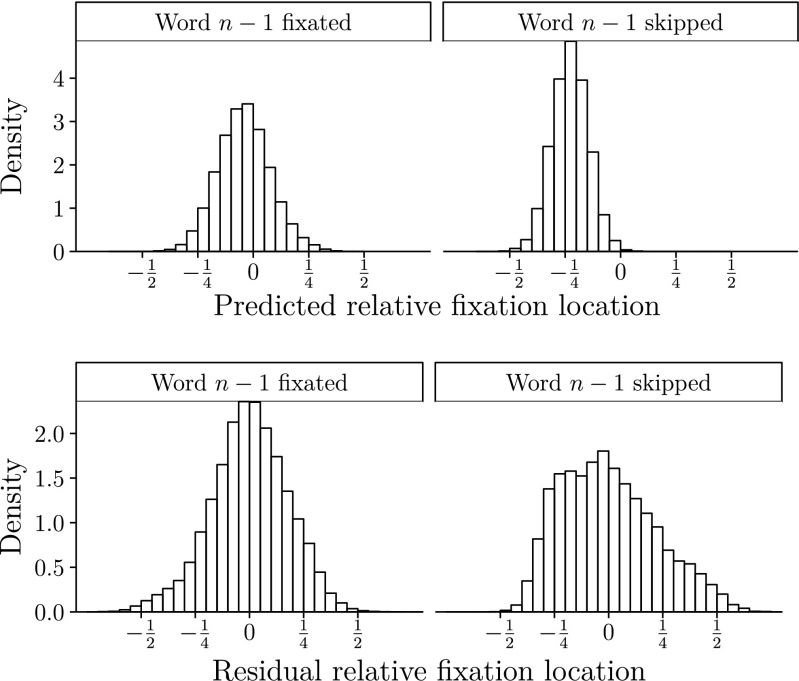
Distribution of predicted values (*upper panels*) and residuals (*lower panels*) of relative fixation location as a function of skipping of word *n*−1

Figure 3 displays the distributions of predicted and residual fixation location. Skipping of word *n*−1 causes a leftward shift of the distribution. Interestingly, some predicted fixation location after skipping saccades are below $-\frac {1}{2}$ indicating the intended word was to the left of the fixated one. Although the distributions of the residuals for skipping and nonskipping cases are slightly skewed, the combined distribution of all residuals follows a normal distribution. Both predictions and residuals provide unique information, as they are uncorrelated by definition (*ρ*≈0.03).

In the first linked LMM, we include *predicted* fixation location (specified as an orthogonal second-order polynomial). In comparison with the model including observed fixation locations, the fit was significantly better (see Table 3). In a further model, we include *residual* fixation location. Again, the fit improves compared to the model with observed fixation location. Finally, we create a third linked LMM with both *predicted and residual* fixation location. This model shows the best goodness-of-fit, indicating that both variables contribute to goodness of fit.

At first glance this may appear to be an astonishing result, because obviously the sum of the predicted and residual fixation locations is simply the observed fixation locations ($x = \hat {x}+\epsilon _{x}$). The fixation-duration LMM, containing both the predicted and residual rather than the observed fixation locations, significantly increases both AIC and BIC (Akaike 1998; Schwarz 1978). The mere increase in the degrees-of-freedom (compare Table 3) cannot explain the increase in goodness of fit because both AIC and BIC penalize an increase in the number of parameters. Rather, it is the structure of the fixation-location model, precisely its ability to decompose the observed fixation locations, that adds new information to the fit of the fixation-duration model. In the following, we focus on the results of the model containing both the predicted and residual values of fixation location as estimated in the fixation-location LMM.

**Fig. 4:**
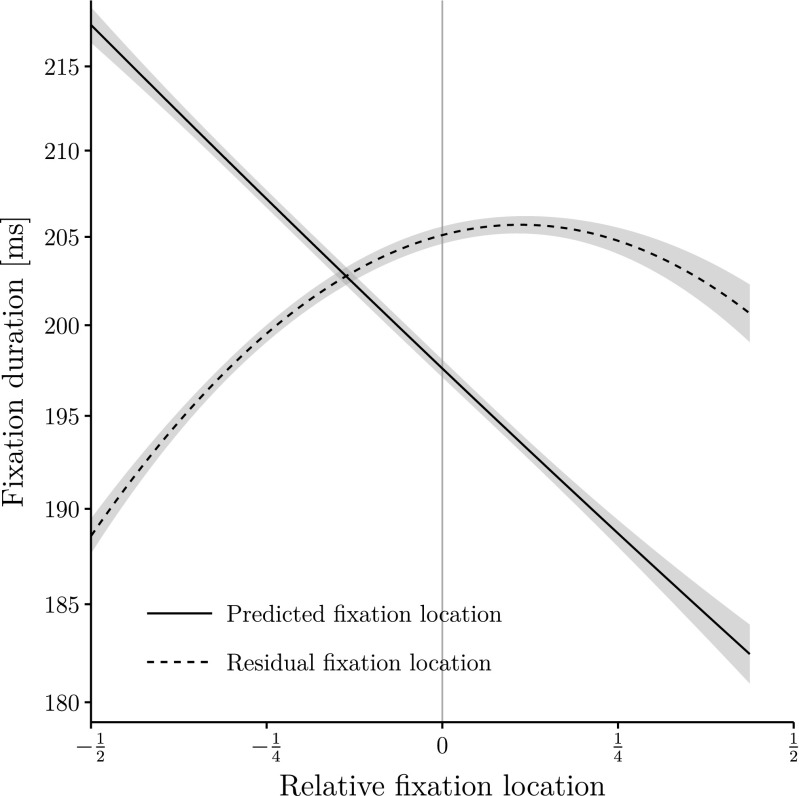
Fixation duration on word *n* (on a log scale) as a function of predicted and residual relative fixation locations together with a second-order polynomial regression curve. The figure displays partial effects created with the remef package (Hohenstein & Kliegl, 2015). Errorbands represent 95 % confidence intervals

Again, there are several significant effects of the properties of word *n*−1, *n*, and *n*+1. The model coefficients are displayed in Table 4. The pattern of effects is very similar to the one of the model with observed fixation locations with two exceptions: The formerly unreliable effects of word length for word *n*−1 and word *n* reach significance. And we obtain different linear and quadratic effects associated with predicted and residual fixation locations! The results are shown in Fig. 4.

The negative-quadratic effect of the observed fixation locations on fixation durations is not reproduced with fixation locations as they were predicted by psycholinguistic word properties in the fixation-location LMM. Actually, there is even a relatively small *positive* quadratic trend associated with the effect of these predicted fixation locations on single fixation durations, indicating an OVP effect. Clearly a very strong negative linear effect dominates the effect of predicted fixation locations on fixation durations, indicating shorter single fixations from the beginning towards the center and end of words.

In contrast, the curve for the effect of the residual of the predicted fixation locations on single fixation durations is similar to the effect obtained for observed fixation locations. Indeed, the significant negative-quadratic effect of fixation duration resembles the IOVP effect. Thus, the classic IOVP effect for fixation durations is not reproduced with fixation locations as predicted by psycholinguistic word properties, but it is reproduced with the residuals of this prediction.

## Discussion

We propose a joint analysis of fixation locations and fixation durations in natural reading, accounting for a large number of effects related to psycholinguistic word properties and a directed effect of fixation location on the subsequent fixation duration at this location. To this end, first we decomposed observed fixation locations into predicted and residual fixation locations estimated in a traditional LMM. Then we used these variables as covariates for the fixation-duration LMM, thereby linking the first model into the second one. As a consequence of this linking, the IOVP effect of fixation location on single-fixation duration was reproduced with residual fixation location. If psycholinguistic word properties have an effect on fixation location, then such a decrease of fixation duration as fixation location shifts from the beginning to the center of a word is predicted by cognitive processing efficiency and is in agreement with results from isolated word recognition. In the following, we discuss these direct effects on fixation location and direct and indirect effects on fixation durations, highlighting the insight gained through linked LMMs.

### Fixation location

#### Visual, oculomotor, and lexical influences

The analysis of fixation locations reproduced known effects of the joint influence of visual and oculomotor variables and further revealed novel results about the impact of linguistic word properties. We replicated the canonical effects of length of words *n*−1 and *n*, launch-site distance, and skipping of the preceding word on fixation location in word *n*. Moreover, we obtained effects of word frequency and predictability on fixation locations. These effects are particularly interesting as they constitute evidence for linguistic influences on saccade programs which materialize as shifts in fixation locations.

The average relative fixation location increases with the predictability of word *n*−1, the origin of the incoming saccade in nonskipping cases. This result indicates that ongoing lexical processes might influence the programming of the saccade goal. Fixating a difficult, low-predictable word results in a shorter saccade than fixating an easy, high-predictable word, presumably because of less remaining resources for the processing of upcoming letters in the parafovea (cf. Henderson & Ferreira, 1990). This psycholinguistic processing difficulty results in shorter saccades and therefore potentially reduces the amount of new information extracted with the following fixation such that more resources remain for finishing ongoing processes. However, the effect is reversed for the frequency of word *n*−1. This result is opposite to the expectation that high processing difficulty leads to right shifts of fixation locations.

The effects of word *n*’s frequency and predictability on the fixation location are positive, as expected by the account in term of local processing difficulty. If the word is easier to process (i.e., it is more frequent or highly predictable), saccades land further into the word. Readers might use parafoveal information of the first letters to obtain the processing difficulty of the upcoming word. If they expect an easy word, the next saccade is longer to allow for processing additional information in the parafovea.

In summary, for three out of four covariates, an increase in processing difficulty was associated with shifts of fixation locations to the beginning of words. In similar analyses of reading of simplified and traditional Chinese script, Kliegl et al. (2014) reported right shifts of fixation locations whenever processing difficulty was low on word *n*−1 or word *n* with respect to frequency, character complexity, or predictability of words. In other words, the direction of twelve covariate effects was in the expected direction. Thus, there remains a bit of a puzzle with respect to the opposite effect of frequency of word *n*−1 on fixation location on word *n*.

The interpretation of the interaction effects between skipping and word properties is straightforward. The impact of the properties of word *n*−1 is stronger if it is skipped. In skipping cases, word *n*−1 is an upcoming word. Not surprisingly, the decision to skip the word and consequently the fixation location are affected by the properties of the to-be-skipped word. In contrast, the effects of the properties of word *n* are smaller if word *n*−1 was skipped. This outcome is plausible too, as the successive word is preprocessed to a larger degree than a word further to the right.

#### Systematic and random variations

In summary, there are numerous effects of visual, lexical, and oculomotor variables on the computation of saccade goals (fixation location) in reading. In addition to the fixed effects, the model for fixation location included several random effects. Based on this complex model, we decomposed observed fixation location into predicted and residual fixation location. Residuals represent deviations of the fixation location that cannot be explained by psycholinguistic covariates or between-subject and -item variability. These deviations can be due to (a) not taking into account other important covariates, (b) nonlinear relationships between covariates and fixation locations, and (c) oculomotor error (imperfections in saccade targeting). Sources (a) and (b) can never be ruled out completely in regression analysis.^5^ However, there is evidence that (c), *random oculomotor error*, contributes to variance between fixation locations (McConkie et al. 1988). Taking into account random error causing deviations from the intended saccade target, fixation locations in reading cannot be predicted perfectly. Indeed, random saccade errors are implemented in computational models of eye-movement control in reading, for example, SWIFT (Engbert, Nuthmann, Richter, & Kliegl, 2005; Risse et al., 2014) and E-Z Reader (Reichle, Pollatsek, Fisher, & Rayner, 1998; Reichle, Warren, & McConnell, 2009), but an inherently random variable cannot be used as a covariate in statistical models.

In the present context, the predicted fixation locations represent intended fixation locations based on several variables, mostly relating to psycholinguistic properties of words *n*−1 and *n*. If fixation locations depend on psycholinguistic properties, the preferred viewing location, based only on word length and possibly launch site, is only part of the story; see Wei et al. (2013) for a similar argument with respect to saccade amplitudes. Rather saccade programs also depend on local processing difficulty. Thus, the intended fixation location appears to depend both on low-level visual and high-level cognitive processes based on current and expected word difficulties. Of course, random deviations from these predicted fixation locations are captured in the LMM residuals.

### Fixation duration

Single-fixation durations were significantly affected by properties of the currently fixated word *n*, the previous word *n*−1, and the upcoming word *n*+1. Since these effects were basically in agreement with previous research (Heister et al. 2012; Kliegl et al. 2006; Kliegl 2007), we will not comment on the results here. Rather we focus on the effect of fixation location on fixation duration and the linking of LMMs.

In the traditional approach with separate LMMs, we used observed fixation location as covariate for fixation duration. As expected, there was a significant IOVP effect: Fixation duration was longest when the eyes fixated near the word center and decreased towards its boundaries.

#### Linking LMMs of fixation location and fixation duration

In a linked LMM, we used predicted and residual fixation locations as covariates for fixation duration. The models were fitted sequentially, such that the results of the fixation-location LMM was incorporated into the fixation-duration LMM. This allowed us to estimate effects of both intended fixation locations (as predicted by mainly psycholinguistic covariates) and random oculomotor error.

Both predicted and residual fixation locations resulted in reliable linear and quadratic effects. Interestingly, the IOVP effect in observed fixation locations was not replicated with fixation locations as predicted by mainly psycholinguistic covariates. The dominant pattern was a linear decrease of fixation duration from beginning to center of words with predicted fixation location. There was also a significant quadratic trend weakening the decrease from the center to the end of words. However, the IOVP effect was obtained with the residuals of these predicted fixation locations. How can one interpret effects of predicted and residual fixation locations?

#### The effect of predicted fixation location

The predicted fixation locations represent intended saccade goals indicating local processing difficulty reflected mainly in psycholinguistic covariates of word frequencies and predictabilities. We observed a decrease in fixation duration with increasing fixation location. Isolated words are recognized most quickly, if they are fixated near their center. This finding does not necessarily need to hold for eye movements in normal text reading. In experiments utilizing the moving-window paradigm (McConkie and Rayner 1975), the *perceptual span* in reading is highly asymmetric, extending much further to the right than to the left of the current fixation location (for a review, see Rayner, 2009). Obviously, the asymmetry of the perceptual span translates into more efficient processing of characters to the right of fixation than characters to the left of fixation. Hence, a fixation location slightly right to the beginning of a word might allow for optimal processing of the fixated word. This is in agreement with the perceptual-economy hypothesis by Vitu et al. (2007). Longer fixations near the beginning of a word are extended due to expected advanced processing. Because of the asymmetry of the perceptual span, fixations near the end of a word may be shorter.

#### The effect of residual fixation location

We observed the IOVP effect only for the residuals associated with the predicted fixation locations. Since residual fixation locations presumably reflect primarily random deviation from the intended saccade goal due to oculomotor error, a value of zero represents a fixation location that is exactly at the intended location. Negative values indicate saccadic undershoot while positive values indicate saccadic overshoot. If the saccade lands near the intended location, fixations are longer compared to too-short or too-long saccades. This observation is in seamless agreement with the hypothesis of mislocated fixations (Nuthmann et al., 2005, 2007).

#### The IOVP effect revisited

Integrating the last paragraphs, we highlight a very specific contribution of our linked LMM analyses to an understanding of eye-movement control during natural reading. The results help to reconcile the paradoxical result that fixation durations are longer in the word center when they should be shorter from a perspective of processing economy and as found in isolated word recognition, that is the IOVP effect. The joint analysis of fixation locations and fixation durations with linked LMMs provides new support for the thesis that the IOVP effect is primarily due to oculomotor errors. More importantly, it appears that the part of fixation location predicted by psycholinguistic properties for the left half of the word is in agreement with results from isolated word recognition: Fixation durations decrease as fixation locations shift from beginning to the center of the word. There is no evidence for an increase of fixation durations as fixation location shifts from center to the end of the word, but this may well be due to the asymmetry of the perceptual span during natural reading; a symmetric perceptual span is quite plausible for the recognition of isolated words that are presented in the center of the screen. In addition, a qualitatively similar dissociation of effects of predicted and residual fixation locations on fixation duration was already found for reading of simplified and traditional Chinese script (Kliegl et al. 2014).

### Linking statistical and computational models

Predictions of fixation durations based on linked LMMs may also provide much more accurate simulation targets for computational models of eye-movement control (e.g., E-Z Reader, Glenmore, and SWIFT) than the table of means for single-fixation, first-fixation, and gaze duration broken down by word frequency and word length. Instead of describing the responses through isolated models for fixation locations and fixation durations, they take into account that both measures are outcomes of entangled processes. They attempt to capture one specific dynamic aspect relating to the relation between local processing difficulty and the selection of the next saccade target.

Computational models differ strongly in how they implement this dynamic aspect. How they do so reflects their theoretical cores. The systematic and graded effects uncovered with linked LMMs present formidable challenges for the next generation of computational models. The qualitatively different effects of predicted and residual fixation location on fixation duration are an example. To the degree that the theoretical assumptions guiding their implementation are correct, the simulations should be able to reproduce such patterns of results at least qualitatively. Possibly, the results are already latently present in the simulation results of some of the computational models.

### Reliability of linked linear mixed models

In order to evaluate the reliability of sequential fitting of linked models, we used simulations to assess the reliability of the regression coefficients of the second linked model (for fixation duration). We sampled values from the distribution defined by the first model (for fixation location) and used the corresponding predictions and residuals to sequentially fit a second linked model. Further technical details of this approach are provided in the supplement. In summary, the obtained coefficients of predicted and residual fixation location (linear and quadratic) were very close to the ones in the original model. The effects remained significant in *all* simulation runs demonstrating the reliability of our sequential linked linear model approach.

There is one serious concern one may have about the two-step approach of estimating a first LMM and then including predicted and residual values of this first LMM as covariates in a second LMM. By linking two LMMs, they actually become one nonlinear mixed model (NLMM). Consequently, this single NLMM should be fitted to the data instead of two LMMs. The fit of a single NLMM provides the advantage that the estimates of the fixed and random effect coefficients associated with the first LMM could be improved through the additional information provided by the observations described by the second model. In the supplement, we demonstrate the concept of a nonlinear joint fit with a simulated data set. It turns out that a joint fit of a single model and a sequential fit of two models produce similar results while the computational costs of the former are much higher. Furthermore, we show that model linking is capable of recovering the simulated linkage of two models whereas separate models fail.

### Linked LMMs beyond eye-movement research

Having to deal with multiple correlated outcomes is probably the default situation for any type of psychological research. These outcomes are usually the result of dynamically entangled processes which we hope to understand via their relation with covariates. Obviously, the application of linked LMMs is not limited to studying the control of eye movements during natural reading. They may be an attractive alternative for the analysis of multiple outcome variables in many other research settings. In contrast to separate statistical models, they capture the redundancy between measures and their relative sensitivity to covariates, making a step forward towards an adequate description of entangled covariates.

Linked LMMs also allow for isolating the indirect contribution of psycholinguistic covariates and thereby testing their influence on critical effects. If a covariate of the first LMM is removed and the model is fitted without this covariate, the variable inherently does no longer affect the predictions. Hence, the contribution of the covariate is shifted from the model predictions to the model residuals. If the covariate does indeed have an effect on a second outcome in a linked LMM, removing the covariate will result in a critical effect of the first model’s residuals.

The decomposition of one outcome into predicted values and residuals, and their use as covariates in a second model, not only allows for the distinction between direct and indirect effects of covariates but also links these formally isolated models. As our reconciliation of different accounts for the paradoxical IOPV effects shows, they even may help us to determine dependencies that remain hidden with separate linear mixed models.

## Electronic supplementary material

Below is the link to the electronic supplementary material.

(PDF 254 KB)

## Footnotes

1
^1^ We use the matrix notation (see also Bates, Mächler, Bolker, & Walker, 2015) for the random effects to reduce the complexity of the formula; they are not of primary interest in the current context.

2
^2^ Substituting $\hat x$ and *𝜖*_*x*_ in Eq. 4 with the corresponding terms in Eq. 1 results in the formula

3
^3^ Due to the inherent correlation between launch-site distance and skipping (the average amplitude of skipping saccades is higher) we also specified a model in which we used orthogonal second-order polynomial terms for launch-site distance. Although this model resulted in a significant interaction between the quadratic trend of launch-site distance and skipping, the general pattern of results matches the one of the model reported here. Furthermore, the influence on linked model fitting is negligible.

4
^4^ The specification of these models with respect to fixed and random effects is the same in all models, with the obvious exception of different fixation-location covariates. These models include variance components for all word-related covariates but not for fixation location because models specifying variance components for all covariates were overparameterized and did not converge.

5
^5^ An additional source of variance, particularly in eye-movement research, is measurement error. While this might contribute to random deviations in the data, differences with magnitudes of the widths of multiple characters are very unlikely when the calibration procedure was performed correctly.
