# Supplementary material for: Linked linear mixed models: A joint analysis of fixation locations and fixation durations in natural reading
Source: Psychon Bull Rev. 2016 Sep 9;24(3):637–51. doi: 10.3758/s13423-016-1138-y (PMC5486867; doi:10.3758/s13423-016-1138-y)
Supplement: Supplementary file 1 — (PDF 254 KB) [file 13423_2016_1138_MOESM1_ESM.pdf]

Supplementary material for: Linked linear mixed models

Sven Hohenstein, Hannes Matuschek, and Reinhold Kliegl
University of Potsdam

June 14, 2016

Overview

In this supplement we report four additional analyses.

Test of ambiguous interaction effects

In this section, we assess the reliability of the interaction effects between skipping and other covariates in the fixation-location model.

Variance components of the LMMs

In this section, we summarize the variance components of the fixation-location model and five fixation-duration models.

The reliability of sequentially fitted linked LMM results

In this section, we address the reliability of the parameter estimates of linkage factors using a simulation based on the fixation-location model.

Joint fit of linked LMMs

In this section, we use simulations to demonstrate (a) the reliability of a sequential fit of linked models (compared to a single joint nonlinear fit) and (b) that model linking is capable of recovering the simulated linkage of two models whereas separate models fail.

Test of ambiguous interaction effects

Table 1 summarizes the results of a test of ambiguous interaction effects as described in Matuschek and Kliegl (2015). Such ambiguous interaction effects may arise when non-linear main effects of dependent covariates are described inadequately (e.g., as linear ones). In these cases, it is possible that an interaction effect between dependent covariates can be explained completely with non-linear main effects. To test for this ambiguity, first we fitted a main-effect-only additive mixed model (AMM) to the data, excluding all interaction effects. Then we fitted a simple linear model to the residuals of the AMM to test whether there are interaction effects that cannot be explained by the non-linear main effects of the AMM.

Table 1
Results of the test for ambigous interaction effects for the landing position model. The estimates and t-values of the LMM estimates are reproduced in the columns Estimate *and* t*. The estimates of these interaction effects after accounting for non-linear main effects are shown in columns* Estimate* *and* t*.

	Estimate	t	Estimate*	t*
Skipping × launch-site distance	0.002	0.466	−0.004	−1.082
Skipping × length (word $n-1$)	−0.050	−1.965	−0.057	−2.652
Skipping × predictability (word $n-1$)	0.013	7.075	0.010	5.704
Skipping × frequency (word $n-1$)	−0.017	−8.239	−0.010	−6.058
Skipping × length (word n)	−0.230	−9.386	−0.135	−6.614
Skipping × predictability (word n)	−0.008	−4.453	−0.005	−3.290
Skipping × frequency (word n)	−0.013	−7.063	−0.009	−5.894

The results were that none of the significant interaction effects found by the landing-position LMM could be explained entirely by non-linear main effects. Except for the insignificant interaction effect of Skipping and launch-site distance, all effect sizes (coefficient estimates) were reduced. Thus, only a small part of these interaction effects can be explained with non-linear main effects; the interaction effects are statistically reliable.

Variance components of the LMMs

This supplement documents the variance components in the models used in the main article. For all models we determined significant variance components. We built the LMMs with the constraint that the models were not overparameterized. In all models, variance components are assumed to be independent. Word properties vary only *between* words, and, therefore, there are no variance components for word properties for the random factor words.

Fixation location

Table 2 lists the square-roots of the variance components of the fixation-location model (i.e., SD). This model ist the first one in the sequence of linked linear mixed models (LMMs). Its predictions and residuals are used as covariates for fixation duration (see below).

Fixation duration

Table 3 lists the square roots of the variance components of the fixation-duration models (i.e., SD). There are five different models with respect to the fixation-location covariate: None, with observed fixation durations, with predicted fixations locations, with residual fixation locations, and with both predicted and residual fixation location. The latter three are linked LMMs as they are based on the fixation-location model. The structure of the variance components is identical for the fixation-duration models.

Table 2
Variance components of the fixation-location model.

Random factor	Variance component	SD
Word	(Intercept)	0.048
Subject	Frequency (word n)	0.012
	Predictability (word n)	0.006
	Length (word n)	0.188
	Frequency (word $n-1$)	0.011
	Predictability (word $n-1$)	0.005
	Length (word $n-1$)	0.168
	Launch-site distance	0.024
	Skipping	0.053
	(Intercept)	0.077
Sentence	(Intercept)	0.028
Residual		0.192

Table 3
Variance components of the fixation-duration models.

		**Fixation location covariate**				
		none	obs.	pred.	res.	pred./res.
Random factor	Variance component	SD	SD	SD	SD	SD
Word	(Intercept)	0.104	0.103	0.105	0.103	0.104
Subject	Frequency (word $n+1$)	0.013	0.013	0.014	0.013	0.014
	Predictability (word $n+1$)	0.010	0.010	0.010	0.010	0.010
	Length (word $n+1$)	0.091	0.091	0.095	0.093	0.098
	Frequency (word n)	0.023	0.023	0.025	0.023	0.025
	Predictability (word n)	0.018	0.018	0.017	0.018	0.017
	Length (word n)	0.319	0.317	0.346	0.318	0.344
	Frequency (word $n-1$)	0.020	0.020	0.018	0.020	0.017
	Predictability (word $n-1$)	0.008	0.008	0.009	0.009	0.008
	Length (word $n-1$)	0.272	0.266	0.217	0.271	0.212
	(Intercept)	0.138	0.137	0.138	0.137	0.137
Sentence	(Intercept)	0.054	0.054	0.055	0.053	0.054
Residual		0.273	0.273	0.266	0.271	0.264

Note. “obs.” denotes “observed”, “pred.” denotes “predicted”, “res.” denotes “residual”.

The reliability of sequentially fitted linked LMM results

To assess the reliability of the estimates obtained with the sequential model-fit approach illustrated in the main manuscript, we ran simulations based on the original data

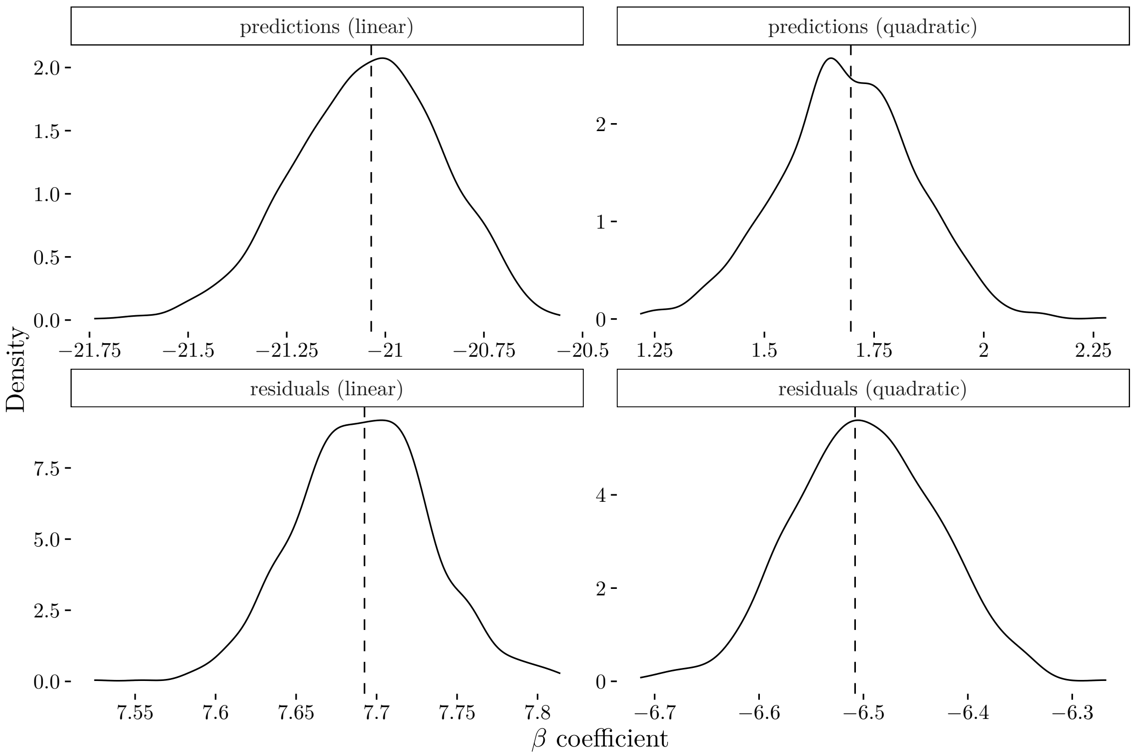

Figure 1. Distribution estimated regression coefficients for predicted values (upper panels) and residuals (lower panels). The solid lines indicate kernel density estimates based on the results of the simulation, the dashed bars indicate the estimates obtained with the original model.

set with more than 80,000 fixations. We sampled deviations from the distribution of the fixed-effect estimates and their variance-covariance structure based on the original fixation-location model. With this, it is possible to determine the uncertainty of the fixation location model predictions and consequently its residuals. To this end, we were able to directly sample new predictions and residuals for the fixation location model without refitting it. In the presence of large datasets, this approach is much faster than, for example, a bootstrap approach. With these surrogate samples for the fixation location predictions and residuals, we refitted the fixation duration model to obtain estimates for the linkage factors. Hence, we obtained multiple results of sequential model fitting.

We sampled $1,000$ independent fixation location predictions and residuals. Beside second-order polynomials for predicted and residual fixation location, the model included further word-related covariates and variance components (see Table 3, columns 1 and 2).

The estimated coefficients turned out to be very close to the original values. All linear and quadratic effects of predictions and residuals remained significant. Figure 1 shows the distributions regression coefficient estimates associated with the second-order polynomials of predictions and residuals. The results of our simulation provide evidence for the reliability of the outcome of sequential model linkage, particularly for large data sets.

Joint fit of linked LMMs

In linked LMMs, the predictions and residuals of the first model can enter the second one as non-linear fixed effects. To this end, it forms a single non-linear mixed model (NLMM). With present implementations of NLMMs (e.g., `lme4`; Bates, Mächler, Bolker, & Walker, 2015), the types of non-linearities that can be modeled are somewhat restricted. For example, the non-linear part of a NLMM in `lme4` can be any function in the fixed-effect coefficients of the NLMM. The non-linear part of a *joint* linked LMM, however, may be a non-linear function in the fixed-effect coefficients, the random-effect coefficients, and in the residuals. Consequently, a joint fit of general linked LMMs cannot be performed by means of present NLMM implementations.

In this section, we simulate simple linked LMMs and demonstrate, that

a) a sequential fit of LMMs is capable of recovering the linkage of the two models reliably, whereas separate fits of independent LMMs fail, and

b) an approximate joint fit reveals almost identical results to the sequential fit of two linked LMMs, while increasing the computational costs for the fit significantlyy.

For this simulation study we sampled artificial data from

$$X_{i,j} = \underbrace{a\,u_{i,j} + s_{x,j}}_{\hat{X}_{i,j}} + \epsilon_{x,i,j} \tag{1}$$

$$Y_{i,j} = b\,v_{i,j} + \alpha\,\hat{X}^2_{i,j} + \beta\,\epsilon^2_{x,i,j} + s_{y,j} + \epsilon_{y,i,j} \tag{2}$$

where $X_{i,j}$ is the i-th response X ($i = 1, ..., 100$) of the j-th *subject* ($j = 1, ..., 30$), $Y_{i,j}$ is defined analogously. The parameters $a = -0.5, b = 0.5$ are the fixed effect coefficients associated with the covariates $u_{i,j} \sim U[-1, 1]\,i.i.d.$ and $v_{i,j} \sim U[-1, 1]\,i.i.d.$, respectively. $s_{x,j} \sim \mathcal{N}(0, 1)\,i.i.d.$ and $s_{y,j} \sim \mathcal{N}(0, 1)\,i.i.d.$ are the random effect coefficients for the j-th *subject*. Parameter $\alpha = 1$ is the linkage factor of the prediction of the model 1 ($\hat{X}_{i,j}$) into model 2, while the parameter $\beta = -1$ is the linkage factor for the residuals of model 1 ($\epsilon_{x,i,j}$) into model 2. Finally, $\epsilon_{x,i,j} \sim \mathcal{N}(0, 1.21)\,i.i.d.$ and $\epsilon_{y,i,j} \sim \mathcal{N}(0, 1)\,i.i.d.$ are the model residuals. Please note that the coefficients a, b, α, β as well as the random effect and residual variances are chosen such that the direct effect of $X^2_{i,j}$ on $Y_{i,j}$ is small.

In general, a joint fit of linked LMMs is difficult as the two samples $X_{i,j}$ and $Y_{i,j}$ are not independent and their dependency is non-linear. The samples $X_{i,j}$ and $\tilde{Y}_{i,j} = Y_{i,j} - \alpha\,\hat{X}^2 - \beta\,\epsilon^2_{x,i,j}$, however, are independent as the residuals $(\epsilon_{x,i,j}, \epsilon_{y,i,j})$ and random effect coefficients $(s_{x,j}, s_{y,j})$ are defined as independent for this simulation. Consequently, the joint likelihood of $X_{i,j}$ and $\tilde{Y}_{i,j}$ given the model parameters α, β, θ_X and $\theta_{\tilde{Y}}$ can be expressed as

$$f(X_{i,j}, \tilde{Y}_{i,j} \mid \alpha, \beta, \theta_X, \theta_{\tilde{Y}}) = f_X(X_{i,j} \mid \theta_X)\, f_{\tilde{Y}}(\tilde{Y}_{i,j} \mid \alpha, \beta, \theta_X, \theta_{\tilde{Y}})\,, \tag{3}$$

where $f_X(\cdot \mid \cdots)$ is the likelihood of the LMM $X_{i,j} = a\,u + s_{x,j} + \epsilon_{x,i,j}$ and $f_{\tilde{Y}}(\cdot \mid \cdots)$ is the likelihood of the LMM $\tilde{Y}_{i,j} = b\,v + s_{y,j} + \epsilon_{y,i,j}$. The latter also depends on the model parameters θ_X, as $\tilde{Y}_{i,j} \mid \alpha, \beta, \theta_X = Y_{i,j} - \alpha\,\hat{X}^2_{i,j} \mid \theta_X - \beta\,\epsilon^2_{x,i,j} \mid \theta_X$ does. Consequently, the model parameters $\theta_X, \theta_{\tilde{Y}}$ as well as the linkage parameters α, β can be estimated my means of maximizing the likelihood in Eq. (3).

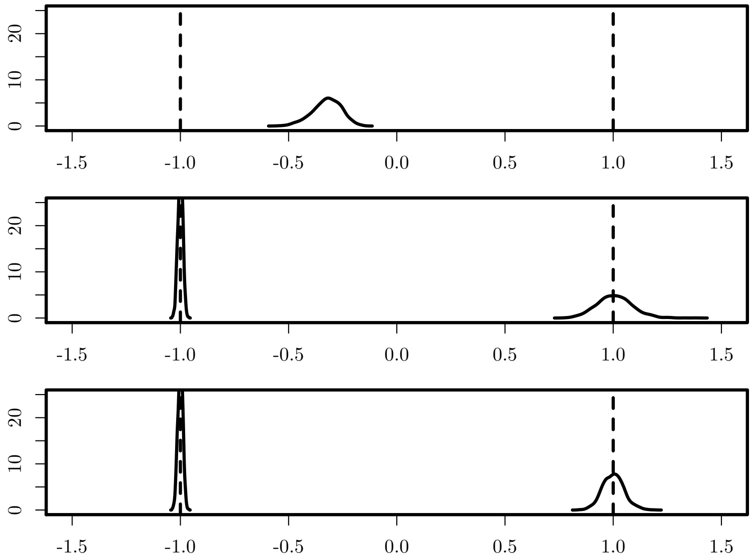

Figure 2. Comparison of the linkage parameter estimates (α, β) as obtained by the direct fit (upper panel, only α) sequential fit (mid panel) and approximate joint fit (lower panel). The vertical dashed bars show the true values of the linkage parameters ($\alpha = 1, \beta = -1$) while the solid lines are the kernel density estimates of the parameter estimates. Whereas the direct fit is not able to recover any of the two linkage effects, the sequential and approximate joint fit are.

Results

For this simulation study, we sampled 1,000 independent datasets from the linked LMMs (Eqs. 1 and 2 above) and fitted three different models to each dataset.

- A direct fit of two independent LMMs, where the second model does not contain the predictions and residuals of the first model as fixed effects. Instead the *observed* $X_{i,j}$ are used as a quadratic fixed effect. That is, $Y_{i,j} = b\, v_{i,j} + \alpha\, X_{i,j} + s_{y,j} + \epsilon_{x,i,j}$.
- A sequential fit of two linked LMMs. As in the article, here we first fit the LMM 1 to the samples $X_{i,j}$. Then, the predictions and residuals of this model $(\hat{X}_{i,j}, \epsilon_{x,i,j})$ are obtained and enter the LMM 2 as quadratic fixed effects.
- An approximate joint linked LMM fit as described above is performed to obtain estimates for the linkage parameters α, β by means of maximum likelihood.

Figure 2 shows the simulation results. It displays the kernel density estimates of the distribution of the linkage parameter estimates. In the top panel of Figure 2, the distribution of the fixed effect of the *observed* response $X_{i,j}$ on $Y_{i,j}$ is shown. It is obvious that the *pure* observations are not able to describe any of the two linkage effects (by construction). It shows a small negative quadratic effect of the *observed* $X_{i,j}$ on $Y_{i,j}$.

The middle panel of Figure 2 shows the distributions of the parameter estimates associated with the quadratic fixed effect of the predictions $\hat{X}_{i,j}$ (α, right) and residuals $\epsilon_{x,i,j}$ (β, left) on $Y_{i,j}$. These estimates were obtained by means of sequentially fitting two

LMMs as in the main article. This approach is able to recover the linkage parameters α, β reliably ($\bar{\alpha}_{seq} = 1.01, \bar{\beta}_{seq} = -1$).

The third panel of Figure 2 shows the same distribution of parameter estimates, but obtained using the joint fit of the linked LMMs as described above. Compared to the sequential fit, the results are almost identical, particularly concerning the β estimates. That is, the estimates of the quadratic effect of the residuals $\epsilon_{x,i,j}$ on $Y_{i,j}$. Although the joint estimation of the quadratic effect of the prediction $\hat{X}_{i,j}$ on $Y_{i,j}$ (α) reveals almost identical results with respect to the means ($\bar{\alpha}_{seq} = 1.01$, $\bar{\alpha}_{join} = 1$), the standard deviation of the joint estimates is slightly smaller than the standard deviation of the sequential estimates ($sd(\alpha_{seq}) = 0.0813$, $sd(\alpha_{join}) = 0.0502$), thus the linkage factor estimates obtained by a joint fit are more reliable than those obtained by a sequential fit. The difference, howerver, is small.

To this end, we conclude that the sequential fit of linked LMMs is a surprisingly good approximation of the joint fit of LMMs by means of maximum likelihood, particularly concerning the expectation value of the linkage parameter estimates. The joint estimation of the linkage parameters, however, is slightly more reliable. Hence the much larger costs for fitting joint linked LMMs compared to the sequential fit might be preferred with small sample sizes or weak linkage.

References

Bates, D. M., Mächler, M., Bolker, B. M., & Walker, S. C. (2015). Fitting linear mixed-effects models using lme4. *Journal of Statistical Software*, *67*(1). doi: 10.18637/jss.v067.i01

Matuschek, H., & Kliegl, R. (2015). *On the ambiguity of interaction and nonlinear main effects in a regime of dependent covariates.* (arXiv:1512.02834 [stat.AP]) Retrieved from `http://arxiv.org/abs/1512.02834`
